# Synthesis and Application of Polymer SXFA in the Detection of Organophosphine Agents with a SAW Sensor

**DOI:** 10.3390/polym16060784

**Published:** 2024-03-12

**Authors:** Cancan Yan, Molin Qin, Tengxiao Guo, Lin Zhang, Junchao Yang, Yong Pan

**Affiliations:** State Key Laboratory of NBC Protection for Civilian, Beijing 102205, China; ccy805905145@163.com (C.Y.); qinmolin@139.com (M.Q.); guotengxiao@sklnbcpc.cn (T.G.); zhanglin_zju@aliyun.com (L.Z.); yangjunchao1990@163.com (J.Y.)

**Keywords:** SXFA, SAW-SXFA sensor, organophosphorus agent, GB

## Abstract

The effective detection of isopropyl methylfluorophosphonate (GB, sarin), a type of organophosphine poisoning agent, is an urgent issue to address to maintain public safety. In this research, a gas-sensitive film material, poly (4-hydroxy-4,4-bis trifluoromethyl)-butyl-1-enyl)-siloxane (SXFA), with a structure of hexafluoroisopropyl (HFIP) functional group was synthesized by using methyl vinylpropyl dichlorosilane and hexafluoroacetone trihydrate as initial materials. The synthesis process products were characterized using FTIR. SXFA was prepared on a 200 MHz shear surface wave delay line using the spin-coating method for GB detection. A detection limit of <0.1 mg/m^3^ was achieved through conditional experiments. Meanwhile, we also obtained a maximum response of 2.168 mV at a 0.1 mg/m^3^ concentration, indicating the much lower detection limit of the SAW-SXFA sensor. Additionally, a maximum response standard deviation of 0.11 mV with a coefficient of variation of 0.01 and a maximum recovery standard deviation of 0.22 mV with a coefficient of variation of 0.02 were also obtained through five repeated experiments. The results show that the SAW-SXFA sensor has strong selectivity and reproducibility, good selectivity, positive detection ability, high sensitivity, and fast alarm performance for sarin detection.

## 1. Introduction

Sarin (GB, methylphosphonic difluoride) is a representative chemical warfare agents. It is an organophosphorus (OP) neurotoxic agent with high volatility, strong toxicity, and a short latency period. This nerve agent can be obtained easily, with characteristics of easy synthesis and difficulty in prevention and control [1,2]. Due to the high specificity and affinity of acetylcholinesterase, GB poses a great threat to human health and public safety [1,2,3]. Therefore, effective detection methods can qualitatively and quantitatively detect GB, improving protection capabilities.

Various gas sensing techniques have been developed for GB detection, for instance, field-effect transistors [4], fluorescence [5], flame photometry [6], ion mobility spectrometry [7], gas chromatography–mass spectrometry [8], and surface acoustic wave (SAW) [9], and each technique has special advantages and plays unique roles in GB detection. The SAW technique has been systematically and deeply studied in the detection of chemical warfare agents (CWAs), mainly due to its non-destructive nature, compact structure, ability to detect nerve agents and blister agents, and applicability to point or area detection [10,11,12,13,14,15]. A SAW sensor has a compact structure and high sensitivity and is small, inexpensive, and capable of fast responses, characteristics that are in line with the current development direction of intelligence in the field of chemical sensors, which are becoming a research hotspot in this field [13,14,15,16]. So far, SAW sensors for detecting various gases, such as H_2_, SO_2_, H_2_S, and NO_2_, have been developed and have yielded remarkable results [16,17], and sensitive film materials play a decisive role in the detection effect [16]. Among the various sensitive film materials, polymers are the most commonly used in SAW sensors [18]. One of these sensitive film materials, SXFA, is an organosilicon compound with a special structure and properties [13,14,19]. Its structural unit has a hexafluoroisopropyl (HFIP) functional group, which has a strong hydrogen-bonding effect on organophosphorus compounds [14,19,20,21]. Thus, it provides superior sensitivity and selectivity in the detection of organophosphorus compounds, and so far, it is one of the most widely studied and data-rich polymers [13,14,20,22,23,24,25]. However, after the synthesis of SXFA, SAW-SXFA sensors generally only exist as sensors for detecting hydrogen-bonding alkaline gases in sensor arrays, and there are few reports on the detection of organophosphorus gases individually.

Therefore, in order to individually detect GB with high sensitivity, we synthesized the polymer SXFA and constructed a SAW-SXFA sensor in this study, discussed its relevant mechanisms, analyzed its detection of organophosphorus nerve agents, and evaluated its practical performance.

## 2. Materials and Methods

### 2.1. Reagents and Instruments

Allydichloromethylsilane, 95%, Macklin, Shanghai, China; phenyltrimethylammonium, 20~25% formaldehyde solution, Tokyo Kasel Kogyo Co., LTD., Tokyo, Japan; hexafluoroacetone trihydrate (HFA·3H_2_O), 95%, Macklin, Shanghai, China; polyepoxypropyl chloride, average Mw ~700, Macklin, Shanghai, China; ether, 99.9%, TEDIA, Fairfield, OH, USA; sulfuric acid, AR, Beijing Chemical Plant, Beijing, China; magnesium sulfate, AR, Macklin, Shanghai, China; toluene, AR, Aladdin, Seattle, WA, USA; ethanol, ≥99.7%, Tansoole, Shanghai, China; dry ice, Yojanbio, Beijing, China; DMMP, AR, Beijing Chemical Plant, Beijing, China; GB, 99%, State Key Laboratory of NBC Protection for Civilian, Beijing, China.

FTS-185 infrared spectrometer, Bio-Rad, Hercules, CA, USA; Q100 modulated DSC, Thermal Analysis, San Diego, CA, USA; Gel Permeation Chromatography (GPC), Waters, Mass, USA; surface acoustic wave oscillator, with central oscillation frequency of 300 MHz and a delay line surface consisting of a quartz layer, State Key Laboratory of NBC Protection for Civilian, Beijing, China; frequency counter, Proteck C3100 (Republic of Korea), Qingdao, China; equipped with RS232 interface for computer connection; Scanning Potentiometer, S4800, Fujifilm, Tokyo, Japan; Dynamic Gas Generator, State Key Laboratory of NBC Protection for Civilian, Beijing, China.

### 2.2. Experimental Methods

#### 2.2.1. Mechanism of Interaction between Polymer and Gas Molecules

A SAW gas sensor utilizes sensitive film materials on piezoelectric crystals to generate characteristic responses for gas adsorption, and polymers are commonly used as the sensitive film materials on its surface [26,27]. Usually, polymers need to have the following characteristics: (1) non-volatility, which can enable polymers to remain stable on sensors for a long time; (2) viscoelasticity, which allows gas to disperse quickly within a polymer film; and (3) quick response capability, selectivity, recoverability, and the ability to be deployed on sensor surfaces [28]. Polymer sensitive film materials are mostly composed of polysiloxane as the main chain, which assumes a viscoelastic state at room temperature and has a good adsorption capacity for gas [29,30]. Before selecting polymer film materials, it is necessary to analyze the principles of interaction between polymer films and gas molecules. Generally, the main interactions between gas molecules and polymer films are van der Waals forces, polarization, and hydrogen bonding [29,30].

**Figure 1:**
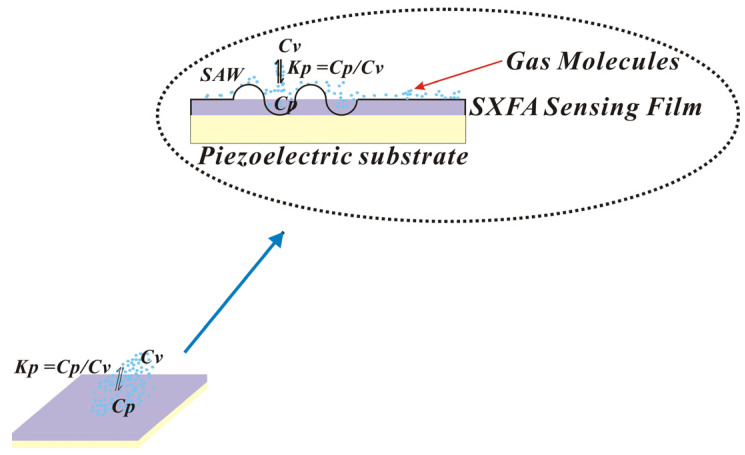
Equilibrium partitioning of vapor molecules between gas phase and polymer.

The process of the polymer adsorption of gas is similar to that of the dissolution of gas into liquid (Figure 1). At the interface between the polymer film surface and the gas phase, target molecules are distributed between the gas phase and the polymer phase, reaching a thermodynamic equilibrium state. Many previous studies have investigated the distribution equilibrium between adsorbate and stationary phases and proposed relevant models [24,25]. The selective adsorption of gas-phase molecules by polymers and the equilibrium between target molecules in the gas phase and the polymer phase can be expressed by the following formula:

*Kp* = *Cp*/*Cv* (1)

where *Kp* represents the equilibrium of gas entering the polymer phase from the gas phase, *Cv* represents the concentration of the target molecule in the gas phase, and *Cp* represents the concentration of the target molecule in the liquid phase. In this model, *Kp* quantitatively describes the equilibrium of gas entering the polymer phase from the gas phase, and a larger *Kp* value represents a stronger gas adsorption capacity.

To determine the *Kp* value, Grate et al. proposed the linear solvation energy relationship (LSER) as a model for the selective adsorption of gases; this model is established by using a gas as a solute and a polymer as a solvent [31]. The solubility of a gas is expressed by a series of parameters, with LESR representing a linear combination of all forces, and the liner relationship is as follows:

LogK = c + r R_2_ + s π _2_^H^ + a α _2_^H^ + b β _2_^H^ + lLogL^16^ (2)

Among them, the dissolution parameters *R*_2_, *π*_2_*^H^*, *α*_2_*^H^*, *β*_2_*^H^*, and *LogL*^16^ represent various solubility parameters of gases: *R*_2_ refers to the gas hyper-molar regression parameter, and this quantitatively represents *n* and *p* electrons, which play a polarization role; *π _2_^H^* represents the gas dipole or polarization parameter; *α*_2_*^H^* and *β*_2_*^H^* represent the parameters of the gas hydrogen-bonding acid and hydrogen-bonding base, respectively; *LogL*^16^ represents the distribution coefficient of the solute between the gas and liquid phases at 25 °C (obtained from gas–liquid chromatography) and the van der Waals force of the gas; *r*, *s*, *a*, *b*, and *l* are related to the properties of the polymer films; *a* and *b*, as supplements to gas hydrogen-bonding acidity and hydrogen-bonding alkalinity, represent the hydrogen-bonding acidity and hydrogen-bonding alkalinity of the polymer films; *s* represents the polarity and dipole effect of the polymer films; *l* represents the dispersion effect of the polymer films, and a larger value of *l* indicates a significant difference in the distribution coefficients of similar gases; *r* represents the polarization ability of *n* and *π* electron pairs between the polymer phase and solute molecules; and *C* is a constant [32].

According to the LSER equation, the interaction between a certain gas and a polymer film can be calculated [33]. Polymers should have sensitivity and selectivity towards the target gas. Additionally, LSER’s polynomial coefficients, such as b/a, b/s, s/a, and l/(s + a + b), can also represent selectivity [34], and Table 1 presents the SXFA polymer’s dissolution selectivity obtained based on the LSER coefficient.

It is clear that, for hydrogen-bonded alkaline gases such as GB, the sought polymer should have the largest possible hydrogen-bonding acidity (b) while having the smallest possible hydrogen-bonding alkalinity (a) and dipole polarity (s), represented by b/a and b/s, respectively. As shown in Table 1, it is clear that, with a strong hydrogen-bonding acidity, the SXFA polymer’s solubility values of b/a, b/s, s/a, l/(s + a + b), and dispersibility are 6.07, 7.08, 1.17, 0.86, 5.55, and 0.13, making it an ideal hydrogen-bonding acidic polymer sensitive membrane material for SAW sensors.

**Figure 2:**
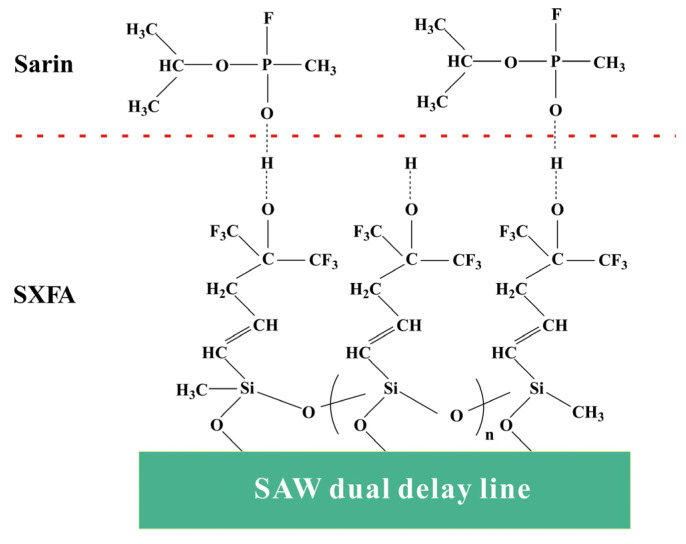
Schematic of sensing mechanism.

Additionally, SXFA is a polysiloxane film material with a low glass transition temperature [14]. The HFIP functional groups on SXFA have strong hydrogen-bonding effects on organic phosphine gases and exhibit the viscoelastic properties of polysiloxane at room temperature, enabling the selective adsorption of organic phosphine compounds [13,14], which further proves that SXFA is an ideal organic phosphine adsorption material. For instance, as shown in Figure 2, the hydrogen-bond acidity of the -OH group on the HFIP functional group was enhanced due to the influence of the neighboring -CF_3_ group, which allowed it to selectively adsorb organophosphorus gases with alkaline hydrogen-bond interactions, achieving the selective adsorption of alkaline organophosphorus compounds; thus, enhanced SXFA is highly susceptible to forming strong hydrogen bonds with GB.

#### 2.2.2. Synthesis Route of Hexafluoro-2-hydroxyisopropyl Polysiloxane

**Figure 3:**
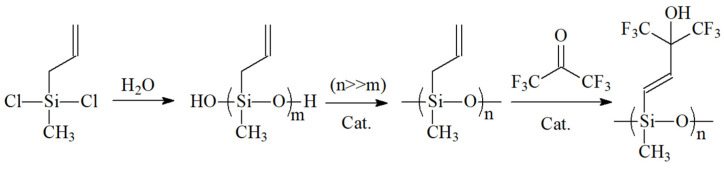
Synthesis route of SXFA.

The synthesis of SXFA requires multiple steps and the control of reaction conditions, so its synthesis method is relatively complex. Figure 3 shows the synthesis steps of the polymer SXFA. In order to ensure the purity and quality of the final product, experimental operations need to be carried out with caution according to the designed process. On the one hand, it should be noted that, in the synthesis process, it is necessary to control the reaction temperature, reaction time, and other parameters. On the other hand, attention should also be paid to the synthesis process to avoid the generation of impurities and by-products.

The first step in the synthesis of SXFA was to prepare methylvinyl polysiloxane by adding an appropriate amount of ether to methylvinyl dichlorosilane, stirring the mixture with a magnetic stirrer, and dripping distilled water on it until complete reaction at room temperature. Then, an appropriate amount of ether was added to extract the upper liquid, and it was dried overnight with MgSO_4_ and filtered. Then, the ether was evaporated, and siloxane was obtained. After the siloxane was prepared, it was left to sit for 3 weeks, and then MgSO_4_ was added for drying (>24 h). It was then filtered, the ether was evaporated, and a small amount of a formaldehyde solution containing phenyl trimethylammonium hydroxide was added. Then, it was stirred at 400 K until the reaction completed and centrifuged to remove the black suspension, and polysiloxane was obtained. Finally, the polysiloxane was transferred to a high-pressure-resistant sealed tube and placed in a dry ice-cold trap. HFA·3H_2_O was dried with H_2_SO_4_, then HFA gas was obtained, and the HFA gas was recovered from the sealed tube in the cold trap. After the reaction was completed, the sealed tube was heated at 380 K (in a silicone oil bath) for 48 h. After naturally cooling, the liquid was removed from the sealed tube and blown with N_2_ overnight to remove the unreacted HFA, and, finally, the final product hexafluoro-2-hydroxyisopropyl polysiloxane (SXFA) was obtained.

#### 2.2.3. Preparation of SAW-SXFA Sensor and Its Detection of Organophosphine Agents

**Figure 4:**
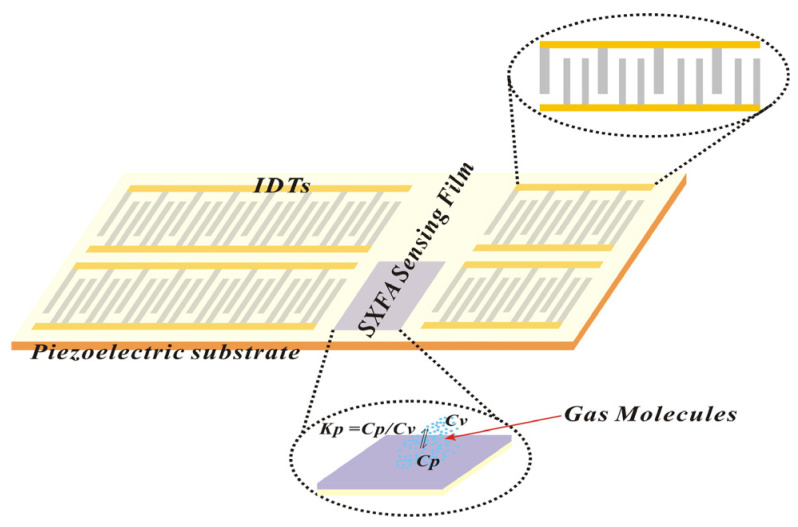
Schematic of SAW-SXFA sensor response mechanism.

The SAW-SXFA sensor consists of an interdigital transducer (IDT), a piezoelectric substrate, and an SXFA gas-sensitive thin film (Figure 4). Due to the piezoelectric effect of the piezoelectric substrate, the input interdigital transducer converts the input electrical signal into an acoustic signal, while the output interdigital transducer converts the received acoustic signal into an electrical signal output. The SXFA film can adsorb gas reversibly on the propagation path of surface acoustic waves, and the increase in its mass leads to a change in the propagation speed of the surface acoustic waves. The detection of gas is achieved by measuring its frequency or phase changes. In this research, we used a delayed linear SAW sensor device with a center frequency of 200 MHz, which was based on Y-shaped quartz cutting. To obtain low-loss and single-frequency signals, unidirectional transducers (SPUDTs) were applied, and structures were combed; the electrode widths of the SPUDTs were ~4 μm and ~2 μm. In the phase detector circuit, the electrical signal emitted by a signal source with a frequency of 200 MHz at a corresponding wavelength of ~15.8 μm and the electrical signal emitted by the SAW-SXFA sensor were output through the phase detector, and a voltage signal proportional to the phase difference of the two signals was then sent to a computer through a data transmission module.

In the preparation of the SAW-SXFA sensor, 150 nm thick aluminum was deposited on the Y-shaped quartz substrate, and a 1 mm thick photoresist was spin-coated and exposed for use in delay line patterns. Then, it was dissolved and rinsed. And, finally, a 50 nm SiO_2_ film was coated on the transducer to provide good protection in the process of the gas-sensitive film coating.

The selectivity of the adsorption of a film is not only related to its structure but also to its morphology. Therefore, in order to improve the separation ability of a sensitive polymer film, the preparation method of thin films can also be controlled. In this study, SXFA polymer films were prepared on sensor components using the drop-coating method to complete the assembly of the SXFA-SAW sensor and applied to detect organophosphine agents.

## 3. Results and Discussions

### 3.1. Infrared Spectroscopy Characterization and Analysis of SXFA Material

During the synthesis process of SXFA, organosilicon compounds have a strong absorption effect on infrared. In this research, the characteristic absorption peaks of the main groups in the synthesis process are Si-Me, Si-CH=CH2, Si-O, Si-O-Si, >SiCl_2_, and -CF_3_ (Si-Me located at ~1260 cm^−1^ and ~765 cm^−1^; Si-CH=CH_2_ located at 1613 cm^−1^, 1410~1390 cm^−1^, 1020~1000 cm^−1^, and 980~950 cm^−1^; Si-OH located at 3390~3200 cm^−1^ and 910~830 cm^−1^_;_ Si-O located at 1100~1000 cm^−1^; Si-O-Si located at 1080 cm^−1^, 1025 cm^−1^, ~1020 cm^−1^, and ~1090 cm^−1^; >SiCl_2_ located at 595~535 cm^−1^; and -CF_3_ located at 1350~1120 cm^−1^, 780~680 cm^−1^, and 680~590 cm^−1^).

**Figure 5:**
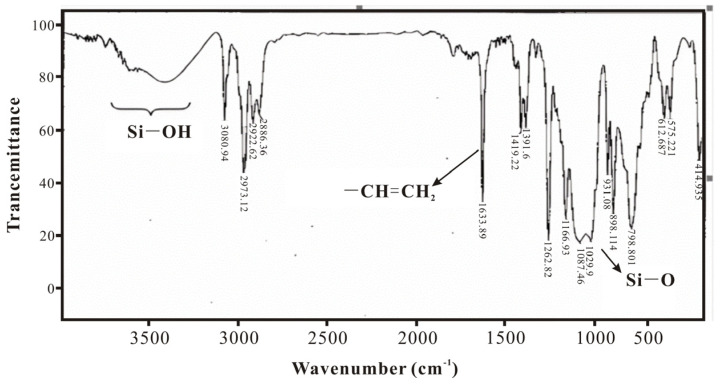
IR spectrum of methylallyldichlorosilane.

Figure 5 shows the IR spectrum of methylvinyldichlorosilane; it is obvious that the peak at 1633 cm^−1^ is the stretching vibration of the vinyl double bond and that the peak at 1263 cm^−1^ is the symmetric deformation vibration of -CH_3_. It can also be observed that there is a Si-OH peak near 3500 cm^−1^ and a Si-O peak at 1080~1025 cm^−1^, indicating that methylvinyl dichlorosilane partially underwent spontaneous hydrolysis and condensation into siloxane. Therefore, methylvinyl dichlorosilane should be stored in a dry environment, and, during the hydrolysis reaction, to ensure the uniform polymerization of siloxane, it should first be dissolved in an organic solvent and then mixed with a suitable amount of water.

**Figure 6:**
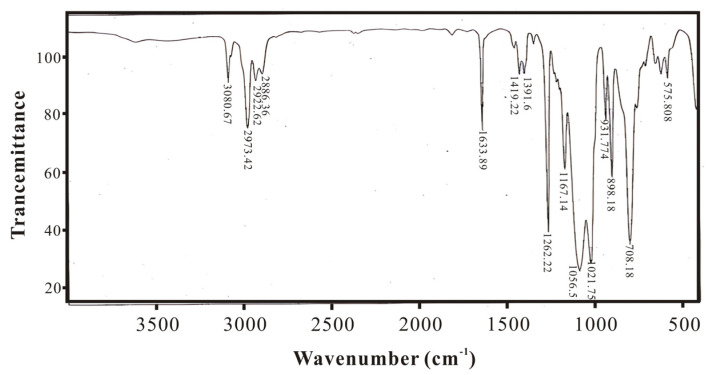
IR spectrum of methylvinylpolysiloxane.

Figure 6 shows the IR spectrum of methylvinylpolysiloxane. Compared with Figure 4, it can be seen that the Si-OH peak near 3500 cm^−1^ disappears, indicating that the raw material was completely hydrolyzed and condensed into siloxane.

**Figure 7:**
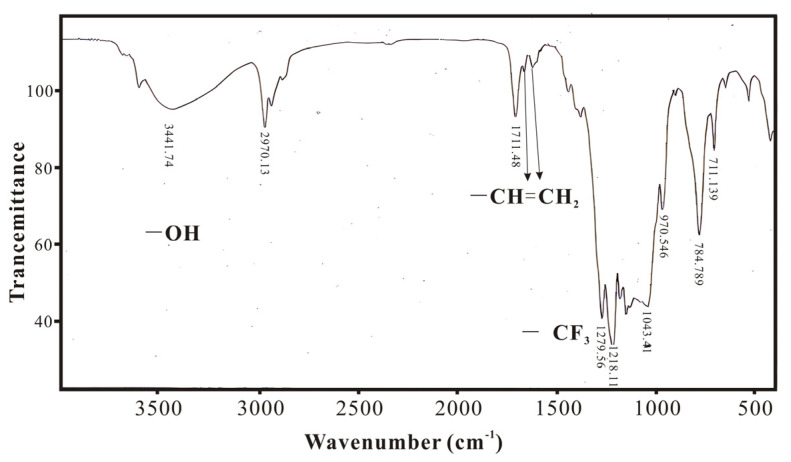
FTIR spectrum of SXFA.

Figure 7 presents the FTIR spectrum of SXFA, which shows the appearance of an -OH peak near 3500 cm^−1^; the original single peak at 1633 cm^−1^ for the double bond has become two adjacent double bond peaks, and there is an -CF_3_ absorption peak between 1120 cm^−1^ and 1350 cm^−1^. This phenomenon indicates that some vinyl double bonds underwent an addition reaction with HFA, causing the double bonds to transfer, and the appearance of hexafluoro-2-hydroxyisopropyl functional group confirms the reaction between methylvinyl polysiloxane and HFA, thus producing the final product SXFA.

### 3.2. SEM Performance

**Figure 8:**
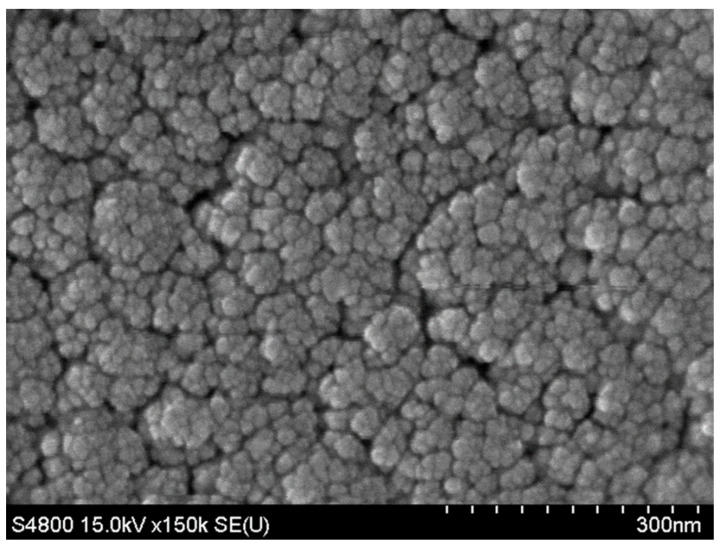
SEM image of SAW sensor delay line of SXFA sensitive film.

SEM performance was used to observe the morphology of the SXFA film on the surface of the SAW sensor and analyze its coverage on the sensor surface. It can be seen in Figure 8 that the SXFA film is in a porous form with a granular arrangement on the substrate, and the polymer has an irregular geometric shape. Therefore, the SXFA film is an amorphous polymer.

### 3.3. Detection of Organophosphorus Agents

#### 3.3.1. Selective Analysis of SAW-SXFA Sensor

**Figure 9:**
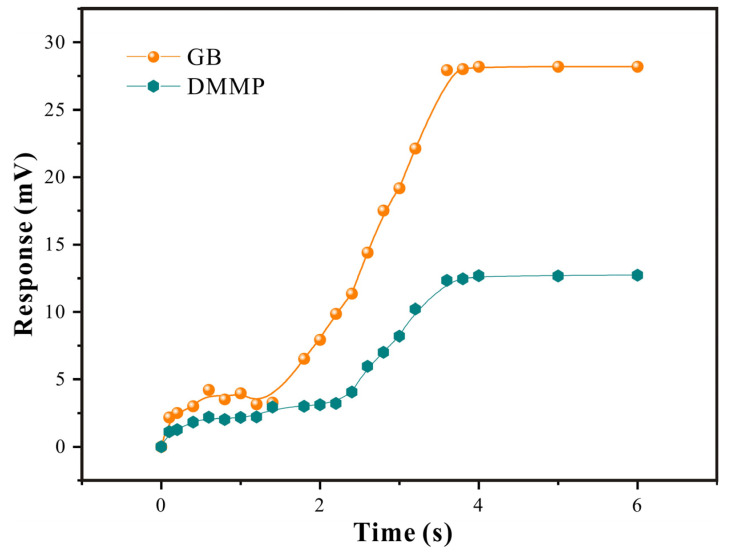
Response of SAW-SXFA sensor with concentration changes in GB and DMMP (19.6 °C, RH = 27%).

**Figure 10:**
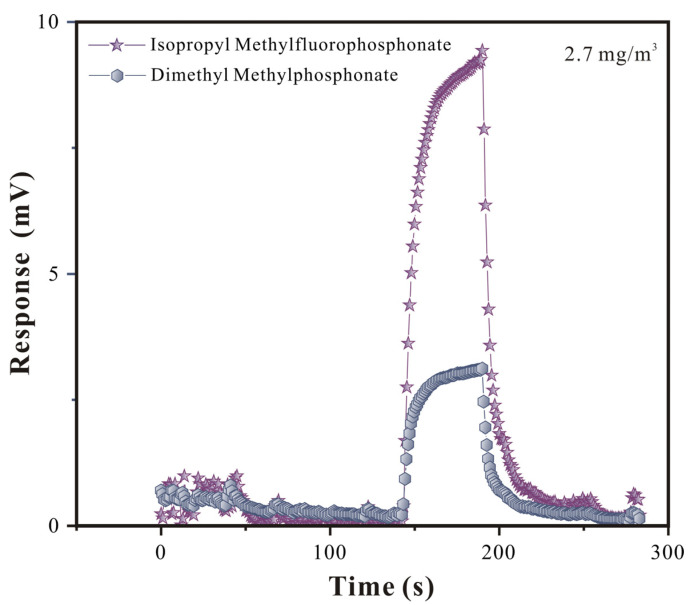
Comparison of GB and DMMP at 2.7 mg/m^3^ (17.9 °C, RH = 28%).

Superior selectivity is extremely important for SAW sensors. So, in this research, a comparative study on the adsorption effect of GB and its analog agent DMMP was conducted (Figure 9 and Figure 10).

In Figure 9, it can be seen that, at low concentrations, the functional group sites on the surface of SXFA were sufficient to adsorb the gas molecules in contact with them, which caused changes in the sensor mass load and thus resulted in significant changes in the sensor signal. As the concentration gradually increased, with the interaction with low concentrations, the functional group sites inside the film interacted with the gas molecules through stereo adsorption and jointly caused changes. However, due to the fact that stereo adsorption was based on the diffusion rate of the gas within the film, the time for the maximum response was prolonged. At a high concentration, both the surface sites and internal sites of the film tended to saturate, so the increase in the GB and DMMP concentrations no longer had a significant impact on the sensor response.

By comparing the responses of the adsorption equilibrium process, it could be seen that the SAW-SXFA sensor had a stronger adsorption response to GB than to DMMP. Initially, the differences in the response gradually increased with the increase in the concentration, and they gradually stabilized when the equilibrium concentration was reached (Figure 9). Through systematic research, it could be illustrated that the main reason for this was that SXFA is a linear polysiloxane-based polymer with an HFIP functional group. The hydrogen-bond acidity of the -OH group on the HFIP functional group was enhanced due to the influence of the neighboring -CF_3_ group, which allowed it to selectively adsorb organophosphorus gases with alkaline hydrogen-bond interactions, achieving the selective adsorption of alkaline organophosphorus compounds [23]. To visually explore the selectivity of the SAW-SXFA gas sensor on GB, response–recovery curves of GB and DMMP at a concentration of 2 mg/m^3^ were compared (Figure 10). As shown in Figure 8, the maximum response of GB was 9.236 mV, while the response signal of DMMP was only 3.124 mV. By comparing the responses of the two toxic gases, it was found that, at this concentration, the adsorption capacity of SXFA for GB was about three times that of DMMP. Therefore, the SAW-SXFA sensor exhibits good selectivity and detection performance for organophosphorus agents.

#### 3.3.2. Analysis of Response of SAW-SXFA Sensor

**Figure 11:**
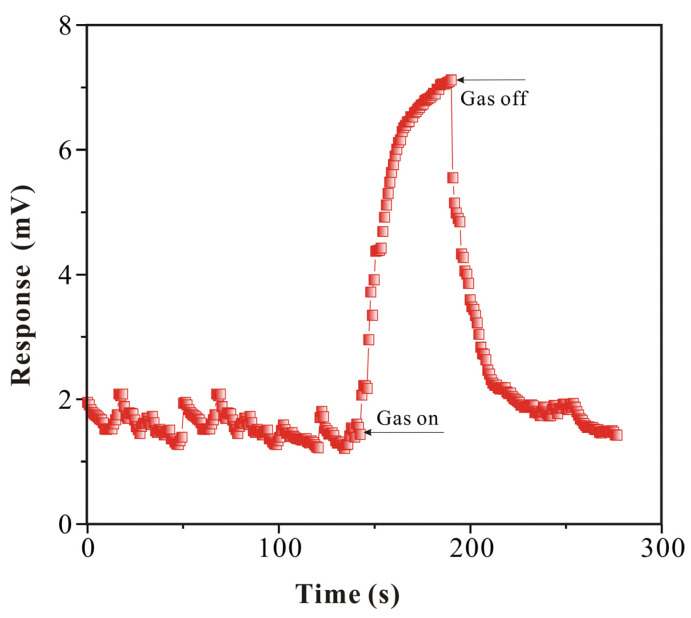
Detection of GB (1.6 mg/m^3^) by SAW-SXFA sensor (18.6 °C, RH = 28%).

The response of gas is crucial in the research of SAW-SXFA sensors. Therefore, relevant research was conducted in this study. In Figure 11, it can be seen that the maximum response of the SAW-SXFA sensor for GB was 6.118 mV, and the noise during the sensor equilibration process could be ignored. At the beginning of detection, due to hydrogen-bond adsorption between GB and the polymer film, the response was 2.475 mV in 10 s, accounting for 40.4% of the maximum response signal, and, furthermore, it only took 50 s to reach 80% of the maximum response. During the recovery phase, GB rapidly separated from the SXFA polymer film, and the response signal decreased rapidly by 4.283 mV within 10 s, accounting for 70% of the maximum response.

In the initial stage, the interactions between GB and the polymer mainly involved the adsorption of surface hydrogen-bonding sites, which indicated a high adsorption efficiency and therefore caused significant changes in sensor mass loading and response signals. After 1 min, some GB molecules penetrated into the interior of the polymer through steric adsorption effects, but the adsorption efficiency at the internal sites of the polymer was lower than that on the surface, so the mass loading of the sensor increased slowly, resulting in a slower response. During the recovery period, the GB molecules on the surface of the SXFA polymer quickly escaped, resulting in a significant change in the sensor signal. Immediately after, with the passage of time, the rate of sensor recovery slowed down, as the GB molecules inside the polymer film had to overcome the obstruction of the polymer chains to escape.

#### 3.3.3. Detection Limit of SAW-SXFA Sensor

The determination of sensor detection limits plays an important role in ensuring the normal operation of sensors, improving usage efficiency, and enhancing safety. In this research, variations in the SAW-SXFA sensor response with the GB concentration were found and are shown in Table 2. When GB was at a relatively high concentration (≥1.0 mg/m^3^), the response signals of the SAW-SXFA sensor showed an increasing trend with the increase in the GB concentration within the same time period, which conformed to the law of solid adsorption isotherms [25], and the SAW-SXFA sensor could recover more than 65% of the response signal. However, when at a relatively low concentration (<1.0 mg/m^3^), the response of the SAW-SXFA sensor increased first and then decreased, and it reached a maximum response at 0.6 mg/m^3^ in this period, similar to the liquid adsorption isotherm curve [25].

**Figure 12:**
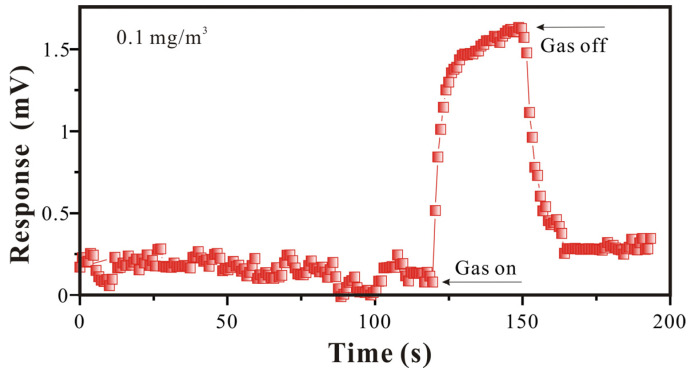
SAW-SXFA sensor for minimum detection concentration of GB (18.6 °C, RH = 29%).

To study the sensitivity of the SAW-SXFA sensor at a low concentration, further analysis was conducted for GB at a concentration of 0.1 mg/m^3^. As shown in Figure 12 and Table 2, the SAW-SXFA sensor response was 1.563 mV in 140 s, and it continued to increase with time. The initial signal change in the response within the first 10 s was 0.326 mV, which was much larger than the SAW-SXFA sensor’s noise, and it could be inferred that the sensor has potential to detect lower concentrations. In addition, it was found that the SAW-SXFA sensor has an “accumulation” function when detecting low concentrations of GB. Under the conditions of a lower sample concentration and a higher alarm response, the SAW-SXFA sensor can achieve a pre-alarm purpose by accumulating adsorption over a long period of time, finally exceeding the alarm limit. Therefore, in the detection of low-concentration GB, the SAW-SXFA sensor has high sensitivity and practical performance as a rapid alarm.

#### 3.3.4. Reproducibility Study of SAW-SXFA Sensor

**Figure 13:**
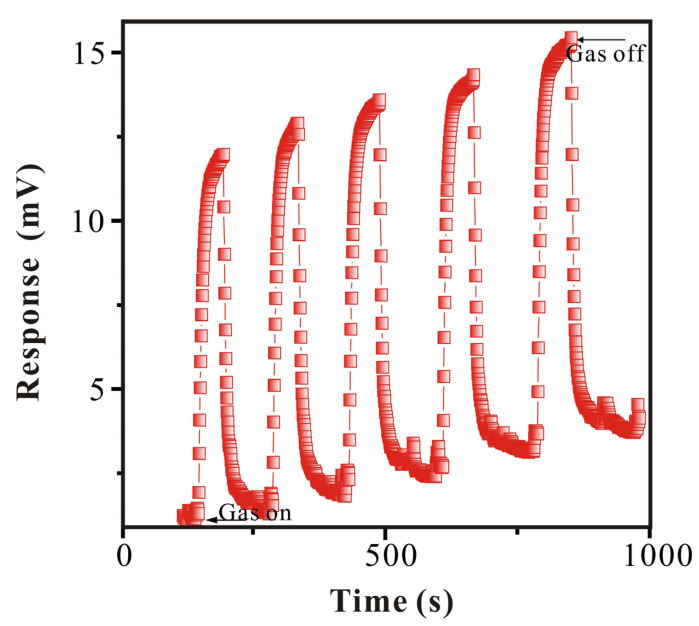
Reproducibility of GB by SAW-SXFA sensor (16.9 °C, RH = 28%).

To research the stability of the SAW-SXFA sensor, GB at the same concentration was detected continuously five times under the same conditions. The response and recovery times were both set to 120 s, and the results are shown in Figure 13 and Table 3.

It can be seen in Figure 13 that the changes in the SAW-SXFA sensor response and recovery times had a periodic nature of about 120 s. However, in this periodic nature of changes, due to the limited recovery time, the GB molecules could not completely desorb from the sensitive film, leading to the continuous accumulation of molecules within the sensitive film, thus resulting in a longer recovery time. Although the SAW-SXFA sensor could not fully recover to the initial value, the impact on the response within the time set in the experiment was very small (as shown in Table 3). The standard deviation of the maximum response measured 5 times was only 0.11 mV, with a coefficient of variation of 0.01, and the standard deviation of the maximum recovery signal was 0.22 mV, with a coefficient of variation of 0.02. Therefore, the SAW-SXFA sensor has good reproducibility for detecting GB, which is of great significance for the future quantitative detection of SAW-SXFA sensors.

#### 3.3.5. Interference Gas Research

**Figure 14:**
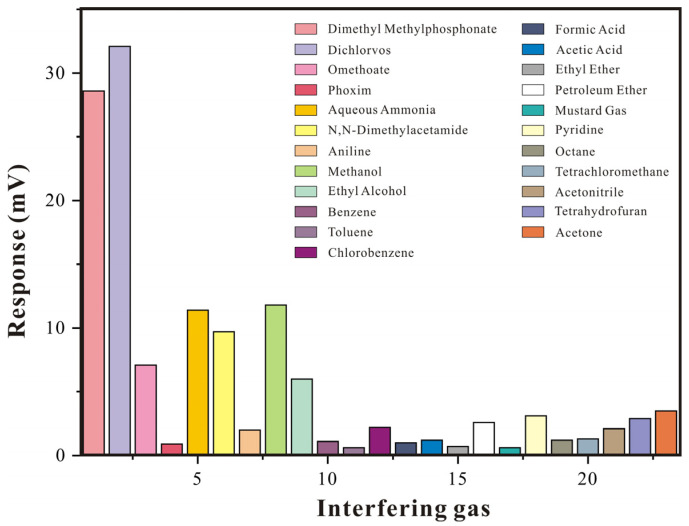
Column chart of interference gas response (19.3 °C, RH = 26%).

The composition of air is quite complex, and for a gas-sensitive sensor, its ability to resist interference is particularly important. Therefore, in order to verify the selectivity of the film material and evaluate sensor performance, it is important to test interference gases before detecting the target gas. The SAW-SXFA sensor was subjected to comparative experiments with various high-concentration interference gases, with each gas concentration set at 500 mg/m^3^, and the results are shown in Figure 14. In Figure 14, it can be seen that the SAW-SXFA sensor had a strong response to organophosphorus gases, especially DMMP and DFP. In addition, due to the hydrogen-bond alkalinity of organophosphorus and amine gases, they could adsorb on the surface of the SXFA polymer through the hydrogen-bond interaction, resulting in a noticeable response to high concentrations of ammonia gas and N, N-dimethylacetamide. Due to the strong polarity of the HFIP group, this group adsorbed various polar gases, causing certain interference of polar gases (such as alcohols) on the SAW-SXFA sensor, and a comprehensive comparison revealed that this effect was much weaker than the hydrogen-bond adsorption effect.

## 4. Conclusions

The synthesis of SXFA and the detection of organic phosphorus gas by a SAW-SXFA sensor were researched in this study, and several conclusions were drawn. Firstly, by analyzing the reaction mechanism and synthesis route of SXFA, it was found that ether plays a dispersing role in dissolving dichlorosilane, resulting in the formation of low-degree cyclic siloxane after hydrolysis and condensation. Phenyltrimethylamine hydroxide has superior catalytic activity, allowing cyclic siloxanes to undergo ring opening and generate chains like polysiloxanes with a high degree of polymerization. HFA has amphiphilic properties and undergoes an addition reaction with methylallyl dichlorosilane, generating HFIP functional groups and transferring the position of allyl double bonds. Secondly, the characterization of SXFA and its relevant materials confirmed that methylallyl polysiloxane reacts with HFA and finally generates SXFA. Then, the detection of GB and its relevant agents indicated that the SAW-SXFA sensor has strong sensitivity (detection limit < 0.1 mg/m^3^), fast response and recovery speed, strong reproducibility and periodicity; additionally, due to its viscoelastic state, its response increases first and then decreases with the increase in the GB concentration at low concentrations, and its maximum response increases with the increase in the GB concentration at high concentrations. And, finally, the interfering gas experiment suggested that the SAW-SXFA sensor has good detection performance and anti-interference ability against organophosphine agents. Although high concentrations of organic phosphines, amines, and alcohol compounds may interfere with sensors, their effects are much weaker than the effects of hydrogen-bonding adsorption.

This comprehensive research suggests that the SAW-SXFA sensor has a good detection effect and interference resistance against organic phosphorus agents and has characteristics such as a short response time, good selectivity, high sensitivity, and strong reproducibility in GB detection, which proves that the SAW-SXFA sensor has superior detection performance for GB.

## Figures and Tables

**Table 1:** SXFA solubility selectivity examined by ratios of LESR coefficients.

Polymer	b/a	b/s	a/s	s/a	b + a + s	Dispersibility
SXFA	6.07	7.08	1.17	0.86	5.55	0.13

**Table 2:** Relationship between concentration of GB and response of SAW-SXFA sensor (17.3 °C, RH = 27%).

Intensity (mg/m^3^)	Response (mV)	Recovery (mV)	Recovery Rate (%)
0.1	2.168	0.02	98
0.2	2.509	0.05	95
0.4	3.002	0.09	91
0.6	4.216	0.08	92
0.8	3.523	0.12	88
1.0	3.972	0.12	88
1.4	3.283	0.14	86
1.8	6.515	0.20	80
2.2	9.859	0.23	77
2.6	14.394	0.27	73
3.0	19.172	0.30	70
3. 4	27.605	0.35	65

**Table 3:** Analysis of data of reproducibility for GB by SAW-SXFA sensor (17.2 °C, RH = 27%).

Experiment No.	Response (mV)	Recovery (mV)
1	10.845	9.696
2	10.619	10.073
3	10.767	10.176
4	10.921	9.729
5	10.831	9.741
Average (mV)	10.797	9.883
Standard Deviation (mV)	0.11	0.22
Discrete Coefficient	0.01	0.022

## Data Availability

Data are contained within the article.

## Dual-use Research Statement

∅ Explanation of Potential Risks: Our paper examines the performance of a SAW sensor using SXFA as a film material. This research is limited to providing some theoretical and experimental support for the development of impact dynamics only and does not pose a threat to public health or national security.
∅ Evaluation of Benefits to the General Public: Our research is limited to the academic field, and it is beneficial for the development of material science. There is no risk to the general public.
∅ Compliance with Laws: As an ethical responsibility, we strictly adhere to relevant national and international laws about dual-use research. And we have considered and adhered to these regulations in our paper.
